# Individualised computerised cognitive training (iCCT) for community-dwelling people with mild cognitive impairment (MCI): results on cognition in the 6-month intervention period of a randomised controlled trial (MCI-CCT study)

**DOI:** 10.1186/s12916-024-03647-x

**Published:** 2024-10-15

**Authors:** Elmar Graessel, Michael Jank, Petra Scheerbaum, Julia-Sophia Scheuermann, Anna Pendergrass

**Affiliations:** Centre for Health Services Research in Medicine, Department of Psychiatry and Psychotherapy, Uniklinikum Erlangen, Schwabachanlage 6, 91054 Erlangen, Germany

**Keywords:** Mild cognitive impairment, Community-dwelling, Computerised cognitive training, Non-pharmacological intervention, Randomised controlled trial

## Abstract

**Background:**

Computerised cognitive training (CCT) can improve the cognitive abilities of people with mild cognitive impairment (MCI), especially when the CCT contains a learning system, which is a type of machine learning (ML) that automatically selects exercises at a difficulty that corresponds to the person’s peak performance and thus enables individualised training.

**Methods:**

We developed one individualised CCT (iCCT) with ML and one basic CCT (bCCT) for an active control group (CG). The study aimed to determine whether iCCT in the intervention group (IG) resulted in significantly greater enhancements in overall cognitive functioning for individuals with MCI (age 60+) compared with bCCT in the CG across a 6-month period. This double-blind randomised controlled study was conducted entirely virtually. The 89 participants were community-dwelling people with a psychometric diagnosis of MCI living in Germany. The iCCT stimulates various cognitive functions, especially working memory, visuo-constructional reasoning, and decision-making. The bCCT includes fewer and simpler tasks. Both CCTs were used at home. At baseline and after 6 months, we assessed cognitive functioning with the Montreal Cognitive Assessment (MoCA). A mixed-model ANCOVA was conducted as the main analysis.

**Results:**

Both CCTs led to significant increases in average global cognition. The estimated marginal means of the MoCA score increased significantly in the CG by an average of 0.9 points (95% CI [0.2, 1.7]) from 22.3 (*SE* = 0.25) to 23.2 (*SE* = 0.41) points (*p* = 0.018); in the IG, the MoCA score increased by an average of 2.2 points (95% CI [1.4, 2.9]) from 21.9 (*SE* = 0.26) to 24.1 (*SE* = 0.42) points (*p* < 0.001). In a confound-adjusted multiple regression model, the interaction between time and group was statistically significant (*F* = 4.92; *p* = 0.029). The effect size was small to medium (partial *η*^*2*^ = 0.057). On average, the participants used the CCTs three times per week with an average duration of 34.9 min per application. The iCCT was evaluated as more attractive and more stimulating than the bCCT.

**Conclusions:**

By using a multi-tasking CCT three times a week for 30 min, people with MCI living at home can significantly improve their cognitive abilities within 6 months. The use of ML significantly increases the effectiveness of cognitive training and improves user satisfaction.

**Trial registration:**

ISRCTN14437015; registered February 27, 2020.

## Background

Mild cognitive impairment (MCI) describes a prodromal stage to dementia [1, 2]. It is characterised by a cognitive decline greater than expected on the basis of age and education, although activities of daily living (ADLs) are not remarkably affected [3]. Thus, MCI refers to a state that is defined by the presence of early cognitive impairments that do not yet constitute a dementia syndrome, along with only very slightly impaired instrumental ADLs [4–6]. People with MCI are at a significantly higher risk of developing dementia than cognitively healthy people, as reported by Inui et al. [1], who found that 72% of patients with amnestic MCI developed Alzheimer’s disease within 5 years. In the general population, the prevalence of MCI increases with age, with rates of 6.7% for ages 60 to 64 and up to 25.2% for ages 80 to 84 [7].

MCI therefore appears to present optimal conditions for interventions that are designed to prevent the transition to dementia (e.g. by delaying the progression of MCI). Research has suggested that there is currently no common effective pharmacological intervention for MCI [7, 8]. For this reason, non-pharmacological interventions have become the focus of research on people with MCI [7–9]. There is evidence that in addition to cognitive activity, the progression of MCI is positively influenced by physical activity and social support [10–14] as well as by other lifestyle factors, such as dietary patterns (e.g. low-fat intake [15]) and socio-economic factors (e.g. high level of education [16]). Modifiable factors that influence MCI can be utilised to develop multimodal non-pharmacological interventions. Hence, various non-pharmacological interventions as well as a combination of interventions may be effective for improving the cognitive abilities of people with MCI [17, 18]. So far, cognitive stimulation has been found to be the most effective intervention [7, 17], followed by physical exercise, multidomain interventions, music therapy, and cognitive training [17]. Wang et al. have described cognitive stimulation as a broad range of activities that provide general stimulation of various cognitive functions and not only training in one specific domain.

Computerised cognitive trainings (CCTs) comprise computer-based tasks that include cognitive exercises, games, and virtual reality. Thereby, users can receive real-time feedback and individualised training [19]. Systematic reviews and meta-analyses of CCT intervention studies have confirmed positive effects of such programmes on the cognitive abilities of people with MCI [2, 20–22].

In a meta-analysis of 18 studies, Zhang et al. [21] reported a small but significant positive effect of CCTs on global cognition as well as on specific cognitive functions compared with the CGs. There were larger effects in studies with a passive CG. The dose and duration of the CCTs varied across the studies, and some of the results of this meta-analysis are difficult to interpret so far. For example, studies with more than 30 h of CCT showed smaller effect sizes than studies with less than ten hours of CCT. However, these results should be interpreted with caution, as only a few studies were included and these were also very heterogenic about the following aspects: the duration of the follow-up period, the variety of instruments used to measure the results, the study designs, the different populations, the insufficient specification of the interventions, the small sample sizes, and the partly non-random allocation of the treatments [23]. In general, the lack of high-quality studies on the effects of CCTs on people with MCI is still a problem.

In order to develop individualised computerised cognitive trainings (iCCTs) that are tailored to the different needs and abilities of participants, research in the field of CCT is currently using more machine learning (ML) and other artificial intelligence (AI) techniques. Instead of standardised cognitive trainings, this personalised approach allows for a more effective adaptation to participants’ individual cognitive performance and creates a tailored learning environment. It can improve the motivation and engagement of study participants who use CCT with the aim of increasing the effect size. Furthermore, they can experience multi-domain benefits, and there is also an improvement in neuroplasticity [24]. However, despite initially promising results [25], the quality of evidence is limited because mostly small, non-randomised pilot studies have been conducted [26]. In CCT for older people with cognitive impairment, AI and more precisely ML methods are pursued to train users at their individual peak performance level. To determine this peak performance, some CCTs exclusively use data on exercise performance [27], but they might also use data from conventional cognitive tests collected through face-to-face testing [28] or mental workload assessed by a non-invasive, optical imaging method for measuring brain activity in the cerebral cortex [29]. Therefore, these approaches rely on externally collected data, so training—and especially the cognitive tests—cannot be performed independently at home. Thus, the CCTs from the aforementioned studies aimed to provide a level of difficulty that fit the current user to increase the efficiency of the training by being challenging without being excessively demanding. To achieve this objective, the researchers used different types of AI.

The basis for the success of any therapy is adherence, that is, that the therapy is also practiced by the person receiving it. Therefore, for CCT to be successful, it is not only important that the cognitive effects have the potential to be achieved, but also that the users are satisfied with the application so that they use the application regularly. For this reason, it is important to analyse user experience in studies on CCT.

As part of this MCI-CCT study, a software application called *MAKSCog* was developed specifically for people with MCI. It is available for computers, laptops, and tablets that can be used at home. A basic CCT (bCCT) was developed as a simpler, comparative intervention (see the ‘Interventions’ section).

### Aim of the study

The present study investigated the research hypothesis that an iCCT (MAKSCog in the intervention group [IG]) leads to statistically significantly greater increases in global cognitive function in people with MCI as compared with bCCT (in the active CG) across a 6-month observation period. We addressed the hypothesis by analysing data on the primary outcome, which was global cognitive function measured by the Montreal Cognitive Assessment (MoCA). Additionally, we evaluated user satisfaction with the iCCT and bCCT.

## Methods

### Study design

A prospective double-blind randomised controlled intervention study (MCI-CCT study) with a 6-month intervention period was conducted to test the research hypothesis. The study participants with psychometrically diagnosed MCI (see the ‘Eligibility of participants’ section) lived in Bavaria, Germany. First patient in was on June 9, 2020; last patient out was on August 5, 2021. Data were collected at baseline (t0) and directly after the 6-month intervention period.

Screening, baseline, and follow-up data (inclusive testing) were collected via videoconferencing and telephone. Participation was voluntary, and participants were free to leave the study at any time without negative consequences. Written informed consent was obtained from all participants. All procedures were approved by the Ethics Committee of the Friedrich-Alexander-Universität Erlangen-Nürnberg (Ref. 58_20B). The study was registered prospectively on February 27, 2020, at ISRCTN registry (trial identification number: ISRCTN14437015). For more information about the study design, see our study protocol by Book et al. [30].

### Recruitment

To recruit study participants, we placed media alerts in two newspapers and one magazine for seniors. In addition, a health insurance company sent a serial e-mail to its insured people aged 60 and older with a reference to the study. Interested individuals could then contact the study centre via e-mail. They were subsequently informed about the project in a personal conversation (videoconferencing or phone call).

### Eligibility of participants

The participants had to fulfil four inclusion criteria in order to be eligible to take part in the study. The first inclusion criterion was that participants were experiencing subjective cognitive decline. People who noticed this decline and who also fulfilled the objective severity criteria for cognitive impairment in the sense of MCI were able to take part in the study. The psychometric level of MCI was defined by a score of 24 points or less on the MoCA (MoCA ≤ 24, step one) and by a score of 24 points or higher on the Mini-Mental State Examination (MMSE ≥ 24, step two). Second, participants had to own a digital device. Third, they had to be at least 60 years old. Fourth, participants had to have signed the informed consent form. Interested study participants were excluded if (1) the technical requirements were not met, (2) the MoCA score indicated no cognitive impairment (MoCA > 24), or (3) the MMSE score indicated dementia (MMSE < 24). An additional reason was if (4) the participants had one of the following impairments: blindness or deafness, acute depression (PHQ-9 > 12), or other psychiatric or neurological disorders that are associated with cognitive decline [30].

### Randomisation and blinding

Blockwise randomisation per recruitment period was performed externally by the Institute of Medical Informatics, Biometry, and Epidemiology (IMBE), Friedrich-Alexander-Universität Erlangen-Nürnberg, only sharing patient/household code, and sex. The latter was used in a stochastic minimisation algorithm to reduce group imbalance [31]. Couples living in the same household were allocated to the same group. The randomisation algorithm was implemented in the statistical software environment R [32].

Data collection at baseline and follow-up was carried out by blinded study assistants. They were psychology students in 3rd year of study with experience in psychometry and had previously received extensive training, specifically in how to administer the primary outcome (MoCA) in a standardized way. The training was conducted by an experienced research assistant and comprised the demonstration and supervision of performance tests, as well as individual feedback and time for questions. Moreover, a detailed manual regarding the standardised execution was distributed to all study assistants.

### Interventions

The development of MAKSCog was based on the cognitive component of the non-pharmacological multicomponent group intervention MAKS® [33, 34], which has been shown to be an effective treatment for people with MCI, mild dementia, and moderate dementia in two independent randomised controlled trials (RCTs) [18, 33, 34]. On the basis of these results, MAKSCog was developed as a CCT that people with MCI can carry out without assistance. It contains an adaptive system, a type of ML that automatically selects exercises at a difficulty level that corresponds to the person’s peak performance and thus enables individualised training. The adaptive system consists of multiple logistic regression classifiers—one for each exercise and difficulty level—which predict the likelihood that an exercise will be successfully completed at a classifier’s level of difficulty, using a cut-off of a 65% success rate. The cognitive status (see the ‘Digital cognitive tests to support machine learning’ section) is used as input for the classifiers. To adapt to the participant, the results of completed exercises are added to the training data in the system, so the classifier is trained once again. Each of the ten tasks of MAKSCog (iCCT) focusses on a different combination of the following cognitive functions—(a) information processing speed, (b) memory span, (c) short-term memory, or (d) decision complexity (reaction to logical reasoning). For additional information, see Book et al. [30]. In addition to MAKSCog, a bCCT with no ML was developed as a control programme. It had fewer and simpler tasks but also stimulated different cognitive functions—preferably requiring long-term memory (e.g. quizzes). Both CCTs could be used completely independently at home without face-to-face contact.

Before baseline testing (t0), the study participants received an email with a link for downloading the software for their version of the CCT. They also received instructions on how to download and install the software. The participants were not told which of the two applications they would be using. In case the installation did not work, technical support was available with detailed and illustrated instructions. Moreover, external technical support was provided during the study period, which was strictly separated from data collection.

## Measures

### Primary outcome

#### Montreal cognitive assessment (MoCA)

The primary outcome, study participants’ cognition at baseline and after the intervention phase (t6), was measured with the Montreal Cognitive Assessment (MoCA) [35]. The MoCA is a valid and reliable screening instrument for assessing MCI [36] and measuring global cognitive functioning [37, 38]. The range of the MoCA score is 0–30 points. We used the cut off of 24 to achieve balance between sensitivity and specificity for MCI (≤ 24) [39–41]. At t0 and t6, different validated parallel versions of the MoCA [42] were used to avoid learning effects in repeated measurements.

### Screening instruments

#### Mini-mental state examination (MMSE)

To screen for dementia, we used the Mini-Mental State Examination (MMSE) [43]. It is the most commonly used screening test for dementia [44]. Scores can range from 0 to 30 points, with higher scores indicating better cognitive performance. Scores between 0 and 23 suggest a dementia syndrome. We decided to apply a conservative cut-off value of 23 in order to minimise the risk of false positive dementia classifications [45–48].

#### Patient health questionnaire (PHQ-9)

To measure symptoms of depression, we used the 9-Item Patient Health Questionnaire (PHQ-9) [49]. The PHQ-9 is a brief self-assessment tool, which is commonly used for depression screening with good reliability and validity [50]. Covering the nine DSM-IV criteria, its nine items ask about experiences over the preceding 2 weeks, rated on a four-point scale ranging from 0 (‘not at all’) to 3 (‘nearly every day’). The total score indicates different degrees of depressiveness. A cut-off score of ≥ 12 demonstrates a good balance between sensitivity and specificity [51].

### Other variables

#### Sociodemographic and health-related data

The sociodemographic and health-related data, modifiable risk factors for MCI, and non-cognitive symptoms were assessed via a standardised questionnaire by student assistants at baseline and follow-up. Sociodemographic data comprised information on age, sex, highest educational level, employment status, monthly income, and household size. Furthermore, as modifiable risk factors for MCI, we assessed general cognitive activities, physical activities, social participation, and nicotine consumption. As health-related data, we assessed diseases (in particular hypertension, hypercholesterolaemia, diabetes mellitus), medications, body weight, and body height.

To assess the effects and side effects of prescribed medication taken by the participants, we used the *medication score* [52]. All effects were ranked from − 2 (strong sedating effect) to + 2 (strong stimulating effect) by two clinical pharmacologists at the Uniklinikum Erlangen, the sum making up the medication score.

To measure the individual vascular risk level, we used the *vascular risk score*. It represents the number of risk factors consisting of smoking, hypertension, hypercholesterolaemia, and diabetes mellitus. The score ranges from 0 (no vascular risk factors) to 4 (four vascular risk factors).

To analyse the comorbidity-related mortality rate, we used the *Charlson Comorbidity Index*, updated and validated by Quan et al. [53]. To form the index, 12 diseases are weighted by 1 (e.g. chronic pulmonary disease) to 6 points (metastatic solid tumour). The sum score ranges from 0 to 24 points. Higher scores indicate a higher one-year comorbidity-related mortality rate, whereby a score of 5 is associated with an 85% 1-year mortality risk.

#### Digital cognitive tests to support machine learning

To assess information about processing speed, memory span, short-term memory, and logical reasoning, the computerised cognitive test battery (CCTB), a collection of digitalised cognitive tests, was developed for this study [54]: if possible, existing valid test items were adapted as digital tests based on, for example, the Wechsler Adult Intelligence Scale (WAIS-IV) [55], the Short Cognitive Performance Test (SKT) [56], the Stroop Test [57], and Raven’s Standard Progressive Matrices [58]. A first pilot study showed satisfactory values for convergent validity [54].

#### User experience questionnaire

To record user experience with the two versions of the CCT (iCCT and bCCT), the study participants were interviewed at the end of the intervention period after 6 months using the User Experience Questionnaire (UEQ) [59]. The UEQ contains 26 items that ask about the characteristics of the programmes in the form of semantic differentials. For this purpose, bipolar adjective pairs are given (e.g. easy to learn–difficult to learn; attractive–unattractive). The respondents provide their judgements on a 7-point scale, which is weighted from + 3 to − 3. Scores are formed for six subscales: attractiveness (6 items), perspicuity (4 items), efficiency (4 items), dependability (4 items), stimulation (4 items), and novelty (4 items). The efficiency, perspicuity, and dependability scales are clustered into the concept of pragmatic quality (usability in the proper sense), whereas the stimulation and novelty scales form the hedonic quality. The items are not ordered by subscales, and half of the items are reverse-scored. Benchmark values based on 452 software evaluations are used to better interpret the results [60]. This procedure allows software to be categorised as excellent (better than 90% of the evaluation results), good (better than 75% of the evaluation results), above average (better than 50% of the evaluation results), below average (worse than 50% of the evaluation results), or poor (worse than 25% of the evaluation results).

### Data collection

The baseline cognition-related parameters (t0 cognitive status) were collected approximately 2 weeks before the other baseline data. These data were collected at the start of the study (via telephone interview to complete the baseline data and transfer the training software) and directly after the end of the intervention (t6). Before the fixed video conference, the study participants were sent the documents for assessing their cognitive status and the questionnaire with socio-demographic and health-related questions via post or e-mail. Parallel MoCA versions were used at t0 and t6 by means of videoconferencing to be performed by trained study assistants. During the remote examination, the study participants were supposed to be located in a quiet and undisturbed room. At t6, the automatically recorded training behaviour was sent on a USB drive to the study centre by post. Parts of the performance tests (i.e. tasks) that the participants needed in a paper pencil version to be able to complete them properly (e.g. visuospatial construction in the MoCA) were sent by post. Thus, the study participants were supposed to open the sealed envelope only in the presence of the tester (i.e. study assistants) at the beginning of the examination. The tester used a standardised evaluation form to make the evaluation. All the completed evaluation forms are being stored safely in accordance with the German data protection guidelines.

### Statistical analysis

To describe the sample characteristics and the group differences, descriptive parameters and the effect size parameters Cramer’s *V* (crosstabs), Cohen’s *d* (*t*-test), and η^*2*^ (ANCOVA) were used. According to Cohen [61], the following limits are used to interpret the effect sizes, which distinguish between small, medium, and large effects: Cramer’s *V* with the limits 0.10, 0.30, 0.50; Cohen’s *d* with the limits 0.20, 0.50, 0.80; and η^*2*^ with the limits 0.010, 0.059, 0.138.

The inferential statistical procedures used were χ^*2*^-tests, *t*-tests, and mixed-model ANCOVAs. Differences between the IG (using iCCT) and CG (using bCCT) for nominally scaled variables were analysed with the χ^*2*^-test and for metric variables with the *t*-test for independent samples. The change in the MoCA score between t0 and t6 was analysed with a *t*-test for dependent samples.

The hypothesis was tested with a mixed-model ANCOVA for the dependent variable MoCA score using the within-subject factor time (t0, t6) and the between-subject factor group (IG, CG) as well as relevant baseline variables as covariates. Relevant covariates had to fulfil two criteria: (A) variables whose preventive influence on the pathological decline of cognition is evidence-based [62] and (B) variables whose difference in the study sample occurred with an effect size ≥ 0.1 (Cramer’s *V* or Cohen’s *d*) between the IG and CG. Thus, potential variables influencing the decline in cognition were included in the evaluation model. To rule out multicollinearity, the covariates were allowed to correlate with each other with a maximum of *p* = 0.80 [63, 64].

To enable the intention-to-treat evaluation, 9 (10%) missing MoCA t6 values (cognitive outcome) were imputed using the expectation maximisation algorithm with group, MoCA score at t0, and all available t0 variables (Table 1) as predictors. Missing values from the UEQ were not imputed.

**Table 1:** Baseline characteristics of the study participants

Variable	Intervention group (*n* = 44)	Control group (*n* = 45)	Group difference (│effect size│)	Total (*n* = 89)
**Sociodemographic**				
Age (years), *M* (*SD*)	73.4 (8.1)	73.5 (6.5)	0.01^$^	73.5 (7.3)
Sex (female), *n* (%)	19 (43.2)	18 (40.0)	0.03^#^	37 (41.6)
Education level			0.08^#^	
Primary school (8–9 years), *n* (%)	4 (9.1)	6 (13.3)		10 (11.2)
Secondary school (10 years), *n* (%)	14 (31.8)	15 (33.3)		29 (32.6)
Higher education (12–13 years), *n* (%)	6 (13.6)	6 (13.3)		12 (13.5)
University degree, *n* (%)	20 (45.5)	18 (0.40)		38 (42.7)
Household income per month (Euro), *M* (*SD*)	3658 (1218)	3644 (1178)	0.01^$^	3651 (1190)
Employed (yes), *n* (%)	5 (11.4)	6 (13.3)	0.03^#^	11 (12.4)
Other persons living in the household (yes), *n* (%)	35 (79.5)	38 (84.4)	0.06^#^	73 (82.0)
**Risk factors for cognitive decline**				
Depressiveness (PHQ-9^a^), *M* (*SD*)	3.1 (2.8)	3.0 (2.8)	0.01^$^	3.1 (2.8)
Vascular risk (sum score^b^), *M* (*SD*)	1.0 (0.9)	1.2 (0.9)	0.13^$^	1.1 (0.9)
Smoking (yes), *n* (%)	3 (6.8)	1 (2.2)		4 (4.5)
Hypertension (yes), *n* (%)	22 (50.0)	25 (55.6)		47 (52.8)
Hypercholesterolaemia (yes), *n* (%)	19 (43.2)	21 (46.7)		40 (44.9)
Diabetes (yes), *n* (%)	2 (4.5)	5 (11.1)		7 (7.9)
Medication score^c^, *M* (*SD*)	0.0 (0.2)	− 0.1 (0.4)	0.19^$^	− 0.1 (0.3)
Cognitive activities (hours per week), *M* (*SD*)	20.8 (13.1)	18.4 (12.0)	0.19^$^	19.6 (12.6)
Physical activities (hours per week), *M* (*SD*)	11.8 (7.3)	9.3 (6.2)	0.37^$^	10.5 (6.8)
Social activities (hours per week), *M* (*SD*)	6.7 (3.8)	7.1 (4.8)	0.10^$^	6.9 (4.3)
Charlson Index^d^, *M* (*SD*)	0.2 (0.4)	0.1 (0.4)	0.06^$^	0.1 (0.4)
Body mass index (≥ 19 and ≤ 25)^e^ (yes), *n* (%)	22 (50.0)	22 (48.9)	0.01^#^	44 (49.4)

^$^Cohen’s *d*

^#^Cramer’s *V*

^a^Patient Health Questionnaire 9

^b^Number of vascular risk factors: range 0 (no vascular risk factor) to 4 (four vascular risk factors)

^c^Sedating (< 0) or stimulating (> 0) effect or side effect of the prescribed medications: sum score of each medication, which was ranked between − 2 (strong sedating effect) and + 2 (strong stimulating effect)

^d^Updated Charlson Comorbidity Index: range: 0–24

^e^Normal range of the body mass index

In the context of the plausibility checks, the follow-up data for the primary outcome MoCA score were analysed for outliers. On the basis of the European Medicine Agency’s guideline’s [65], statistically deviating values were analysed for the observed outlier effect for medical reasons. If a statistically abnormal value (1.5 times the interquartile range) could be explained clinically, this case was excluded from further analyses. The main analysis was carried out without outliers. Results are also reported with outliers.

The analysis of user experience operationalised with the UEQ is primarily descriptive, in particular through the use of standard values [60] and by indicating the effect size of the differences between the bCCT and iCCT.

The analyses were based on an alpha error of 0.05 as the significance level for the main analysis (ANCOVA) and conducted with SPSS (version 28.0).

## Results

### Screening process

**Fig. 1:**
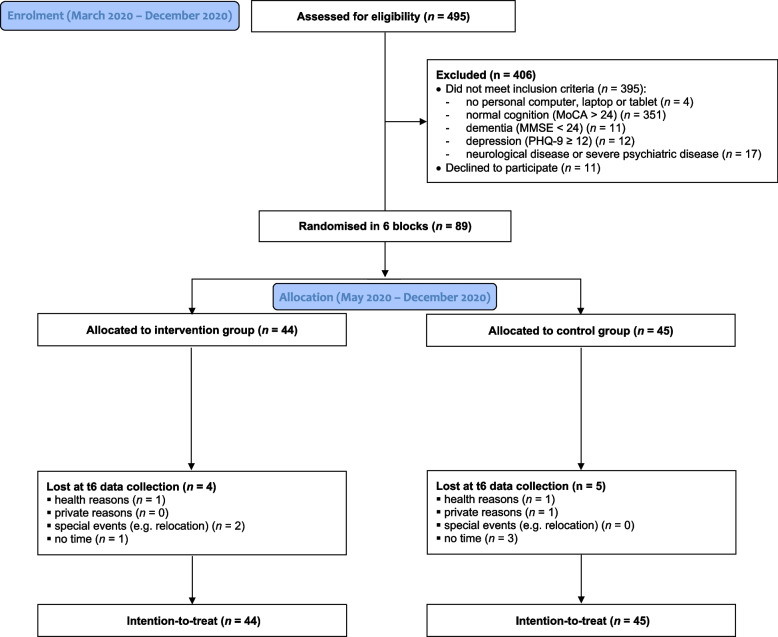
CONSORT flow chart of the MCI-CCT study

Over a period of 10 months, 495 people aged 60 or older living at home were screened for eligibility (Fig. 1). Most people (*n* = 351, 86.5%) had to be excluded because they did not fulfil the MCI criterion psychometrically. Over a period of 8 months, 89 people, randomised into six blocks, were allocated to IG (*n* = 44) or CG (*n* = 45). After 6 months, at the end of the observation period, 40 people in each group were tested and interviewed. The drop-out rate of 10% was equally distributed between IG and CG (4 and 5 people, respectively). As no individuals had died, the intention-to-treat sample comprised all 89 study participants (Fig. 1).

### Sample characteristics

**Fig. 2:**
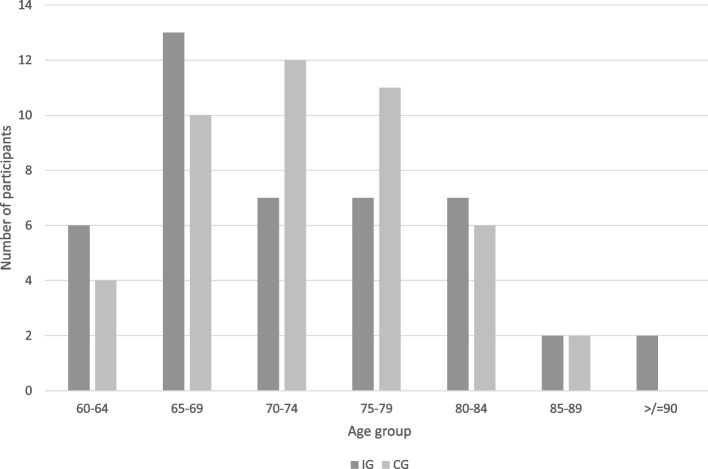
Study participants’ age groups

The 89 study participants were on average 74 years old, and 42% of them were women (Table 1). Figure 2 presents a detailed distribution of the participants by age group. The proportion of 43% with a university degree indicates that the educational level of the study participants was above average.

The study participants were asked about all diseases at baseline. The information was grouped according to the International Classification of Diseases (version 10 [66]). The three most common comorbidity groups were Diseases of the circulatory system (Chapter IX) in 58 people (65%); Endocrine, nutritional, and metabolic diseases (Chapter IV) in 54 people (61%); and Diseases of the musculoskeletal system and connective tissue (Chapter XIII) in 20 people (22%). The 1-year mortality risk was very low in the study population (Table 1), as only 11 people (12%) had Charlson comorbidity scores higher than 0.

Most of the effect sizes (13 of 14) for group differences (Cohen’s *d* or Cramer’s *V*) were less than 0.20 for Cohen’s *d* or less than 0.10 for Cramer’s *V* (Table 1). Only physical activities were higher on average in the IG (11.8 h per week) than in the CG (9.3 h per week; Cohen’s *d* = 0.37). A total of three statistical outliers were identified—two cases in the IG and one case in the CG. A medical cause—infected with COVID-19 combined with impaired cognition according to self-report—was identified for only one outlier in the IG with a deterioration in the MoCA score of 6 points. This case was therefore excluded from further main analyses.

### Exercise duration

As measured in the exercise programmes, the study participants in the IG used the iCCT an average of 15.6 times (*SD* = 6.9) in the first month and 12.7 times (*SD* = 5.6) in the last period. In the CG, the values for the bCCT were 13.7 (*SD* = 6.0) for the first month and 12.3 (*SD* = 6.4) for the last month. The average duration of exercise per day of usage in the first month was 33.9 min (*SD* = 11.8) in the IG and 37.7 min (*SD* = 12.1) in the CG. In the last month, the average duration of exercise per day of usage was 33.6 min (*SD* = 10.8) in the IG and 37.4 min (*SD* = 20.9) in the CG. The total duration of exercise during the intervention period of 6 months was on average 44.1 h (*SD* = 24.1) in the IG and 43.2 h (*SD* = 24.9) in the CG. The difference was small (│Cohen’s *d*│ = 0.04) and not significant (*p* = 0.849).

### MoCA scores in the intervention and control groups

At baseline, cognitive abilities were 0.3 points (MoCA score) higher in the CG than in the IG (Cohen’s *d* = 0.20). The MoCA score increased significantly in both groups during the intervention period—in the CG (bCCT) by an average of 1.0 point (95% CI [0.3, 1.7]) from 22.3 (*SD* = 1.5) to 23.3 (*SD* = 2.4), *p* = 0.008; in the IG (iCCT) by an average of 2.0 points (95% CI [1.3, 2.8]) from 22.0 (*SD* = 1.8) to 24.0 (*SD* = 2.9), *p* < 0.001. According to Cohen’s *d* the increase in the CG (Cohen’s *d* = 0.41) can be classified as small, whereas the increase in the IG (Cohen’s *d* = 0.82) is large. Including the one statistical and clinical outlier, the increase in the average MoCA score in the IG from 22.0 (*SD* = 1.8) to 23.9 (*SD* = 3.1) was also significant (*p* < 0.001) with a Cohen’s *d* of 0.68.

### Main analysis

For the main analysis, five baseline variables met the criteria to be included as covariates (in particular effect size ≥ 0.1; Table 1): physical activities per week (Cohen’s *d* = 0.37), cognitive activities per week (Cohen’s *d* = 0.19), medication score (Cohen’s *d* = 0.19), sum score of vascular risk factors (Cohen’s *d* = 0.13), and social activities per week (Cohen’s *d* = 0.10). No correlations above 0.8 were found between the covariates. The strongest correlation was found between social activities and physical activities (*r* = 0.33). Multicollinearity could therefore be ruled out. After controlling for these five covariates (see Table 2), there was a statistically significant interaction between time and group (*F* = 4.92; *p* = 0.029). The effect size was small to medium (partial η^2^ = 0.057). There was also a small, non-significant main effect of time (*F* = 2.10; *p* = 0.153; partial η^2^ = 0.025). In the analysis in which the outlier O1 was included, the interaction effect between time and group was reduced to *F* = 3.10 (*p* = 0.082, partial η^2^ = 0.036).

**Table 2:** Mixed-model ANCOVA with MoCA score as outcome

	*n* = 88^§^			*n* = 89		
Effect	***F***	***p***	**Partial *η***^**2**^	***F***	***p***	**Partial *η***^**2**^
T^$^	2.10	0.153	0.025	1.09	0.299	0.013
T x cognitive activities*	0.26	0.612	0.003	0.52	0.472	0.006
T x physical activities*	0.43	0.512	0.005	0.24	0.628	0.003
T x social activities*	0.46	0.499	0.006	0.40	0.528	0.005
T x vasc. risk^+^	0.08	0.778	0.001	0.09	0.766	0.001
T x medication^#^	0.23	0.637	0.003	0.29	0.591	0.004
T x group^●^	4.92	**0.029**	0.057	3.10	0.082	0.036

^§^Outlier O1 excluded

^$^T: time

^*^Recorded in hours per week

^+^vasc. risk: number of vascular risk factors: range 0 (no vascular risk factor) to 4 (four vascular risk factors)

^#^medication: medication score is the sedating (< 0) or stimulating (> 0) effect or side effect of the prescribed medications: sum score of each medication, which was ranked between − 2 (strong sedating effect) and + 2 (strong stimulating effect)

^●^group: IG versus CG

Significant *p*-values are printed in bold

**Fig. 3:**
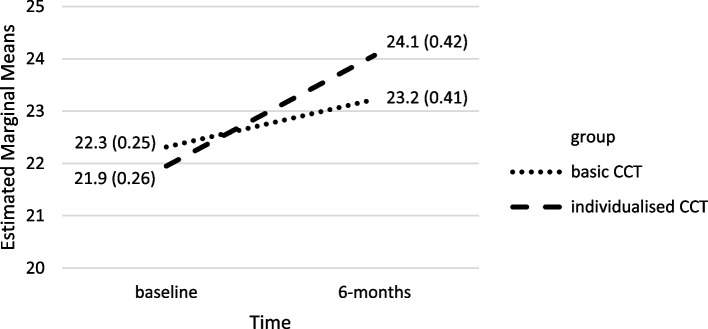
Estimated marginal means of the MoCA score in intervention group (iCCT) and control group (bCCT). Estimated marginal *means* (*standard errors*). Covariates appearing in the model were calculated at the following values: cognitive activities: 19.7 h per week, physical activities: 10.6 h per week, social activities: 7.0 h per week, sum score of vascular risk factors: 1.1, medication score: − 0.1

Figure 3 presents the estimated marginal means and Standard Errors (*SEs*) for the Group x Time interaction (MoCA score). The increase in the IG of 2.2 points (95% CI [1.4, 2.9]) in comparison with the increase of 0.9 points (95% CI [0.2, 1.7]) in the CG met a more extreme level of significance (*p* < 0.001 versus *p* = 0.018).

### Transition rates

In the sensitivity analysis, the transition from MCI (MoCA score ≤ 24) to a better cognitive condition (MoCA score 25–30, not defined as MCI) in the primary outcome (MoCA score) was considered. At baseline, all study participants were in the MCI range (MoCA score ≤ 24, inclusion criterion). After the 6-month intervention phase, 13 out of 45 study participants (29%) achieved a better cognitive condition with the bCCT (CG) and 22 out of 43 (51%) with the iCCT (IG). The difference between groups was statistically significant (χ^2^ = 4.55; *p* = 0.033). When the outlier O1 (IG) was included, the between-group difference was still significant (χ^2^ = 4.16; *p* = 0.042).

### Research hypothesis

Due to the statistically significant difference between the IG and CG in the main analysis (ANCOVA), which was confirmed by the result in the sensitivity analysis, the research hypothesis was supported: The iCCT (IG) led to statistically significant greater increase in global cognitive function over the  6 months observation period in people with MCI compared with the bCCT (CG).

### User experience

**Fig. 4:**
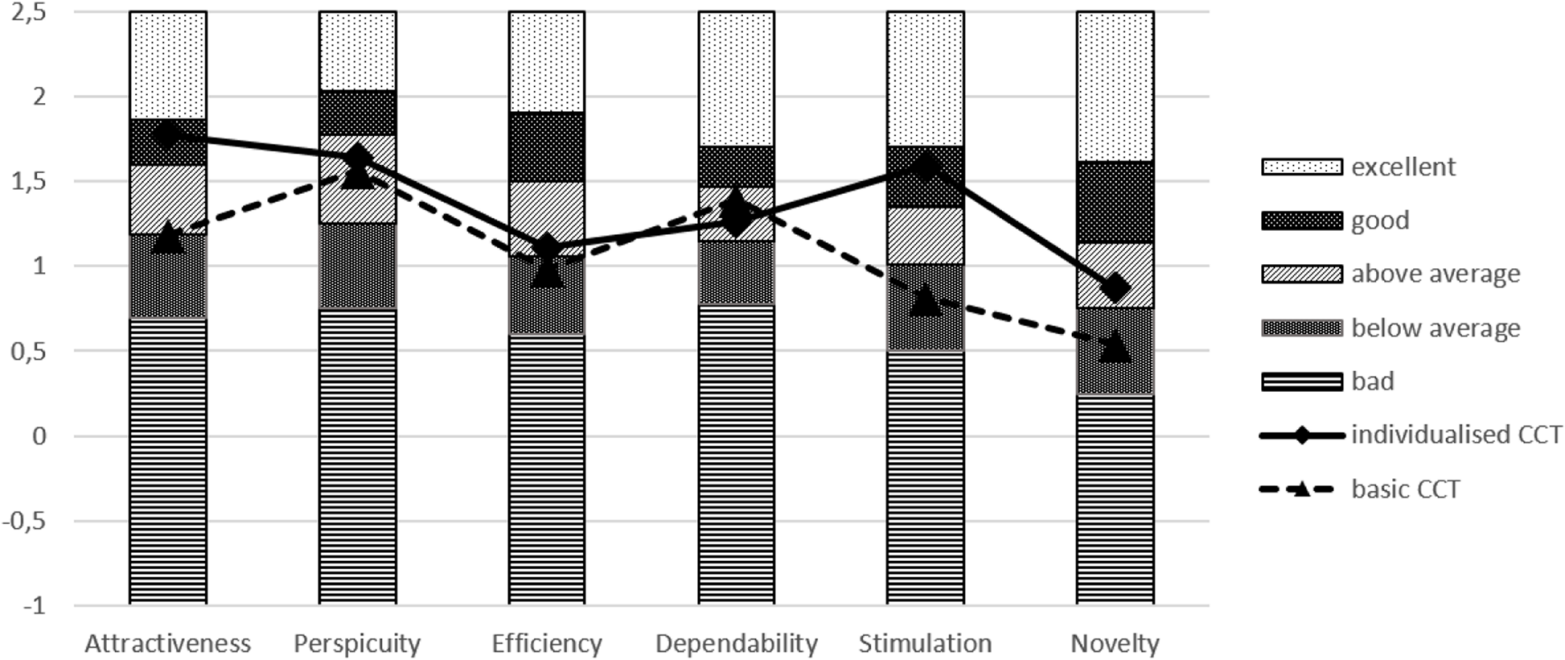
User experience of the two versions of the computerised cognitive training (CCT) classified by the User Experience Questionnaire. Construction of a benchmark for the User Experience Questionnaire (UEQ), see Schrepp et al. [54]; CCT, computerised cognitive training

The user experience results were based on the information provided by 79 study participants: 40 from the IG and 39 from the CG. We classified the UEQ scores (Table 3) according to the general benchmark (Fig. 4).

**Table 3:** User Experience Questionnaire (UEQ) scores in both groups

UEQ dimension	IG *M* (*SD*)	CG *M* (*SD*)
Attractiveness	1.77 (0.81)	1.18 (1.02)
Perspicuity	1.64 (1.03)	1.56 (1.01)
Efficiency	1.11 (0.67)	0.98 (0.63)
Dependability	1.25 (0.74)	1.39 (0.81)
Stimulation	1.59 (0.79)	0.91 (1.29)
Novelty	0.87 (0.91)	0.54 (1.32)

The stimulation and attractiveness scales showed higher values for the iCCT (IG), with a medium effect size for the group difference (stimulation: Cohen’s *d* = 0.73, *p* = 0.002; attractiveness: Cohen’s *d* = 0.64, *p* = 0.006). Small effect sizes, with higher values for the iCCT (IG), were found for the novelty (Cohen’s *d* = 0.29; *p* = 0.200) and efficiency (Cohen’s *d* = 0.20; *p* = 0.371) dimensions. The group differences were not meaningful (Cohen’s *d* < 0.2) for the dependability (Cohen’s *d* = 0.17; *p* = 0.464) and perspicuity (Cohen’s *d* = 0.08; *p* = 0.729) dimensions.

## Discussion

The main focus of this study was on improving the cognition of the study participants with MCI with our newly developed CCTs. Both training programmes—the iCCT with an integrated learning system and the bCCT—led, on average, to significant increases in global cognition with a significant and clinically relevant effect size (partial *η*^2^ = 0.057) for the greater increase in the IG (iCCT) in comparison with the CG (bCCT). It should be noted that spontaneous remissions in the MCI syndrome to cognitively normal test values cannot be excluded. In studies with an observation period of more than 2 years, Canevelli et al. [67] found remission rates of between 18 and 26%. Two facts must be taken into account when interpreting this result. Firstly, depending on the cognitive test used, a varying proportion of false positive results at baseline must also be taken into account, i.e. a proportion of people who therefore had no MCI at baseline. Secondly, the observation period in our study was only 6 months, so that the time-dependent remission rate should be assumed to be lower in comparison with the Canevelli study.

On average, the participants used the training programmes three to four times per week with an average duration of 34 to 38 min per application. For the most part, they did so as recommended—three times per week for 30 min each time. Participants using the iCCT evaluated their programme as more attractive and also felt more stimulated than the participants using the bCCT.

Due to the unexpected onset of the COVID-19 pandemic at the beginning of this RCT, we had to convert all steps of the study into virtual mode. Because of this conversion, we had to drop one of our previously planned additional outcome measures on cognition, the SKT, and we had to rely solely on the MoCA score [30].

As a consequence of the virtual, computer-based study design, only people who had the technical equipment and operating skills to use a computer, laptop, or tablet were included. However, as this technical equipment was necessary for carrying out the CCTs anyway, the online data collection did not result in any fundamental changes to the study design or participant recruitment. However, this technical precondition in combination with the inclusion criterion of age 60 + could explain why the level of education and household income of the study participants were high on average, with 89% of participants having a higher level of education than the basic qualification in Germany. In the total population, this value is 61% [68]. We did not adjust the MoCA score by age, gender, or education [69], but we used a relatively low MoCA cut-off (≤ 24 and not ≤ 26, see O’Caoimh et al. [39]) for the psychometric definition of MCI to reduce the probability of including people who were false positives, especially in a population with a relatively high level of education.

In this study, the result of the block-type randomisation of the participants into the two groups can be described as successful. The median of the effect sizes for the difference between the iCCT group and bCCT group at baseline (including the MoCA at baseline) had a value of 0.03 for Cramer’s *V* (5 variables) and 0.12 for Cohen’s *d* (10 variables).

With both CCTs, we found that participants’ average increase in global cognition was significant. This finding agrees with results from Zhang et al. [21] recent systematic review and meta-analysis, which focussed on effects of CCT on cognitive outcomes in MCI. In the eleven RCTs they included, they found a significant benefit of CCT on global cognition, but the pooled effect size of 0.23 was substantially smaller than the effect size we found in our study, especially in the IG with iCCT (Cohen’s *d* = 0.82). However, the interpretation should be treated with caution because not only the studies but also the instruments they used were very different in this comparatively young field of MCI research. For example, most studies used the MMSE to measure global cognitive abilities, in which the tasks to be solved were actually too easy for the sample of people with MCI and therefore not sensitive enough. Therefore, we used the much more sensitive MoCA test as the primary outcome variable [70, 71], for which an online version already existed [72].

In the studies evaluated in the systematic review by Zhang et al. [21], the effect sizes of studies with active CGs were smaller than of studies with non-active CGs. Although we had an active CG, the increase in global cognition from the iCCT with an underlying learning system was considerably larger than the increase from our bCCT (Cohen’s *d* = 0.4). In general, the effect sizes we identified were high compared with other interventions that have focussed on global cognition in people with MCI, as we found that the MoCA score increased significantly on average by 2.0 points (95% CI [1.3, 2.8]) in the IG with iCCT. For example, Xu et al. [14] evaluated and compared the effectiveness of 17 different types of pharmacological or non-pharmacological RCT interventions in improving global cognition among patients with MCI. Despite the diversity of interventions, only cognition-based interventions (*MD* = 0.80, 95% CI [0.04, 1.57]), physical exercise (*MD* = 1.92, 95% CI [1.19, 2.64]), combined physical exercise and cognition-based intervention (*MD* = 1.86, 95% CI [0.60, 3.12]), and antioxidants (*MD* = 0.94, 95% CI [0.04, 1.83]) had positive effects on global cognition measured with the MMSE in participants with MCI. This finding was taken into account in the analysis in so far as several lifestyle factors—including cognitive, physical, and social activities, which could moderate the effect of the training—were included in the ANCOVA as covariates.

In the treatment of MCI, a fundamental distinction must be made between pharmacological and non-pharmacological therapeutic approaches. The standardised mean difference for the six-month treatment with the iCCT compared with the bCCT was 0.41. For the monoclonal antibodies lecanemab and donanemab—approved in the US—this value was 0.19 and 0.24 respectively, across a treatment period of 18 months compared with a placebo [73].

According to the systematic review by Contreras-Somoza et al. [22], people with MCI can carry out CCT, and most of them provide positive evaluations of the application. The results with regard to user experience with the CCTs we developed showed that elderly people with MCI were able to use CCT and were satisfied with the application, which was crucial for carrying out the intervention. The participants rated the iCCT and the bCCT with rates that were above average in two of three usability scales: perspicuity and dependability. In efficiency, the rates were higher for the iCCT in comparison to the bCCT. However, the differences were small enough, so that no significant or relevant difference in the intensity of use could be observed. The study participants rated the attractiveness and hedonic quality of the iCCT higher than the bCCT. Such perceptions could influence motivation for long-term use. Given that the attractiveness and stimulation of the iCCT received good ratings, and novelty was rated as above average, we assume that the IG study participants will continue to use the cognitive training for longer time periods than the CG participants will continue to use bCCT. Future studies should test this hypothesis.

### Strengths and limitations

Several methodological strengths and limitations were identified. Because the study was conducted remotely, the participants were able to participate regardless of their mobility or their place of residence. However, the need for computers meant that people with a higher level of education were more likely to be targeted and included. Although the self-assessment of cognitive decline was used in the definition of MCI, we did not have a proxy rating that could be applied for a detailed verification of the MCI diagnosis. Furthermore, no individual normative scores that were adjusted for age, gender, or education were used in the psychometric assessment of MCI. As a result, some MCI cases among the people with higher education were not included in the study, but it cannot be completely ruled out that some of the more highly educated study participants already had mild dementia. The validity of the main analysis is enhanced by the elaborate evaluation design, which considered several lifestyle factors as potential moderators of the primary outcome of global cognition. Due to the COVID-19-related contact restrictions and the absence of validated remote test batteries, detailed psychometric performance tests could not be used. In this situation, no clinical data supporting the diagnosis (i.e. no biomarkers) were assessed. In addition, the psychometric assessment of MCI was carried out using screening procedures. However, the testing of the primary outcome and the other data could be completely blinded as part of the remote testing by trained student assistants. The external validity of the results was supported by the fact that the study participants were able to decide completely by themselves when to carry out the therapy thanks to the independent and autonomous implementation of the training programme. On the other hand, the associated unguided implementation of the CCT meant that no direct measures could be taken to ensure adherence (e.g. as compared with training in a rehabilitation clinic). The fact that the CCT was used regularly and for a long time indicates the good experience users had with the application. Furthermore, the results are limited due to the comparatively high educational level of the participants and the small sample size. Thus, the results should be replicated in future studies using a larger sample, in which an attempt should also be undertaken to reach people with a lower educational level. Finally, it must be taken into account that—due to the study design with an active control group—no comparison with the spontaneous, untreated course of MCI was possible.

## Conclusions

This study showed that by using a multi-tasking CCT three times a week for 30 min per session, people with MCI living at home show a significant increase in their cognitive abilities within 6 months. For this reason, digital cognitive training can be used for the secondary prevention of further deterioration in cognitive abilities in MCI as early as possible and due to its uncomplicated application at home it should reach as many people as possible. This training should be easy to use and designed to be diversified, playful, and with many different exercises at different levels of difficulty in order to minimise boredom during prolonged use and to improve user satisfaction. ML should be integrated in order to optimise the effect size and the satisfaction of the users by individualising the difficulty levels of the individual exercises.

## Data Availability

Please contact the corresponding author with any reasonable requests for access to limited, fully anonymised data.

## Abbreviations

ADLs: Activities of daily living
bCCT: Basic computerised cognitive training
CCT: Computerised cognitive training
CG: Control group
CI: Confidence interval
iCCT: Individualised computerised cognitive training
IG: Intervention group
MCI: Mild cognitive impairment
ML: Machine learning
MMSE: Mini-Mental State Examination
MoCA: Montreal Cognitive Assessment
PHQ-9: 9-Item Patient Health Questionnaire
RCT: Randomised controlled trial
SD: Standard deviation
SE: Standard error
UEQ: User Experience Questionnaire

## References

[1] Inui Y, Ito K, Kato T. Longer-term investigation of the value of 18F-FDG-PET and magnetic resonance imaging for predicting the conversion of mild cognitive impairment to Alzheimer’s disease: a multicenter study. J Alzheimer’s Dis. 2017;60(3):877–87. 28922157 10.3233/JAD-170395 PMC5676852

[2] Hu M, Wu X, Shu X, Hu H, Chen Q, Peng L, et al. Effects of computerised cognitive training on cognitive impairment: a meta-analysis. J Neurol. 2019;268:1680–8. 31650255 10.1007/s00415-019-09522-7

[3] Gauthier S, Reisberg B, Zaudig M, Petersen RC, Ritchie K, Broich K, et al. Mild cognitive impairment. Lancet. 2006;367(9518):1262–70. 16631882 10.1016/S0140-6736(06)68542-5

[4] Nygard L. Instrumental activities of daily living: a stepping-stone towards Alzheimer’s disease diagnosis in subjects with mild cognitive impairment? Acta Neurol Scand Suppl. 2003;179:42–6. 12603250

[5] Petersen RC. Mild cognitive impairment as a diagnostic entity. J Intern Med. 2004;256(3):183–94. 15324362 10.1111/j.1365-2796.2004.01388.x

[6] Winblad B, Palmer K, Kivipelto M, Jelic V, Fratiglioni L, Wahlund LO, et al. Mild cognitive impairment- beyond controversies, towards a consensus: report of the international working group on mild cognitive impairment. J Intern Med. 2004;256(3):240–6. 15324367 10.1111/j.1365-2796.2004.01380.x

[7] Petersen RC, Lopez O, Armstrong MJ, Getchius TSD, Ganguli M, Gloss D, et al. Practice guideline update summary: mild cognitive impairment: report of the Guideline Development, Dissemination, and Implementation Subcommittee of the American Academy of Neurology. Neurology. 2018;90(3):126–35. 29282327 10.1212/WNL.0000000000004826 PMC5772157

[8] Kasper S, Bancher C, Eckert A, Forstl H, Frolich L, Hort J, et al. Management of mild cognitive impairment (MCI): the need for national and international guidelines. World J Biol Psychiatry. 2020;21(8):579–94. 32019392 10.1080/15622975.2019.1696473

[9] Yao S, Liu Y, Zheng X, Zhang Y, Cui S, Tang C, et al. Do nonpharmacological interventions prevent cognitive decline? a systematic review and meta-analysis. Transl psychiatry. 2020;10(1):19. 32066716 10.1038/s41398-020-0690-4 PMC7026127

[10] Heser K, Wagner M, Wiese B, Prokein J, Ernst A, König H-H, et al. Associations between dementia outcomes and depressive symptoms, leisure activities, and social support. Dement Geriatr Cogn Disord extra. 2014;4(3):481–93. 10.1159/000368189 PMC4296229 25685139

[11] Guure CB, Ibrahim NA, Adam MB, Said SM. Impact of physical activity on cognitive decline, dementia, and its subtypes: meta-analysis of prospective studies. Biomed Res Int. 2017;2017:13. 10.1155/2017/9016924 PMC5320071 28271072

[12] Cheng S-T. Cognitive reserve and the prevention of dementia: the role of physical and cognitive activities. Curr Psychiatry Rep. 2016;18(9):85. 27481112 10.1007/s11920-016-0721-2 PMC4969323

[13] Levy S-A, Smith G, De Wit L, DeFeis B, Ying G, Amofa P, et al. Behavioral interventions in mild cognitive impairment (MCI): lessons from a multicomponent program. Neurotherapeutics. 2023;19(1):117–31. 10.1007/s13311-022-01225-8 PMC9130435 35415779

[14] Xu Z, Sun W, Zhang D, Chung VC-H, Sit RW-S, Wong SY-S. Comparative effectiveness of interventions for global cognition in patients with mild cognitive impairment: a systematic review and network meta-analysis of randomized controlled trials. Front Aging Neurosci. 2021;13: 653340. 34220484 10.3389/fnagi.2021.653340 PMC8249717

[15] Hou J, Jiang H, Han Y, Huang R, Gao X, Feng W, et al. Lifestyle influence on mild cognitive impairment progression: a decision tree prediction model study. Neuropsychiatr Dis Treat. 2024:271–80. 10.2147/NDT.S435464 PMC10871141 38371917

[16] Caffò AO, Spano G, Tinella L, Lopez A, Ricciardi E, Stasolla F, et al. The prevalence of amnestic and non-amnestic mild cognitive impairment and its association with different lifestyle factors in a South Italian elderly population. Int J Environ Res Public Health. 2022;19(5): 3097. 35270789 10.3390/ijerph19053097 PMC8910691

[17] Wang L-Y, Pei J, Zhan Y-J, Cai Y-W. Overview of meta-analyses of five non-pharmacological interventions for Alzheimer’s Disease. Front Aging Neurosci. 2020;12: 594432. 33324194 10.3389/fnagi.2020.594432 PMC7723835

[18] Straubmeier M, Behrndt E-M, Seidl H, Özbe D, Luttenberger K, Gräßel E. Non-pharmacological treatment in people with cognitive impairment—results from the randomized controlled German day care study. Dtsch Arztebl Int. 2017;114(48):815–21. 29249224 10.3238/arztebl.2017.0815 PMC5752975

[19] Faucounau V, Wu YH, Boulay M, De Rotrou J, Rigaud AS. Cognitive intervention programmes on patients affected by mild cognitive impairment: a promising intervention tool for MCI? J Nutr Health Aging. 2010;14(1):31–5.20082051 10.1007/s12603-010-0006-0

[20] Ge S, Zhu Z, Wu B, McConnell ES. Technology-based cognitive training and rehabilitation interventions for individuals with mild cognitive impairment: a systematic review. BMC Geriatr. 2018;18(1):213. 30219036 10.1186/s12877-018-0893-1 PMC6139138

[21] Zhang H, Huntley J, Bhome R, Holmes B, Cahill J, Gould RL, et al. Effect of computerised cognitive training on cognitive outcomes in mild cognitive impairment: a systematic review and meta-analysis. BMJ Open. 2019;9(8): e027062. 31427316 10.1136/bmjopen-2018-027062 PMC6701629

[22] Contreras-Somoza LM, Irazoki E, Toribio-Guzmán JM, de la Torre-Díez I, Diaz-Baquero AA, Parra-Vidales E, et al. Usability and user experience of cognitive intervention technologies for elderly people with MCI or dementia: a systematic review. Front Psychol. 2021;12:636116. 33967901 10.3389/fpsyg.2021.636116 PMC8100576

[23] Gates NJ, Vernooij RW, Di Nisio M, Karim S, March E, Martinez G, et al. Computerised cognitive training for preventing dementia in people with mild cognitive impairment. Cochrane Database Syst Rev. 2019;3:CD012279. 30864747 10.1002/14651858.CD012279.pub2 PMC6415132

[24] Chapman SB, Aslan S, Spence JS, Hart JJ Jr, Bartz EK, Didehbani N, et al. Neural mechanisms of brain plasticity with complex cognitive training in healthy seniors. Cereb Cortex. 2015;25(2):396–405. 23985135 10.1093/cercor/bht234 PMC4351428

[25] Adolphe M, Pech M, Sawayama M, Maurel D, Delmas A, Oudeyer P-Y, et al. Exploring the potential of artificial intelligence in individualized cognitive training: a systematic review. 2023. 10.1371/journal.pone.0316860 PMC12173387 40526639

[26] Tolks D, Schmidt JJ, Kuhn S. The role of AI in serious games and gamification for health: scoping review. JMIR Serious Games. 2024;12:12(e48258). 38224472 10.2196/48258 PMC10825760

[27] Eun S-J, Kim EJ, Kim JY. Development and evaluation of an artificial intelligence–based cognitive exercise game: a pilot study. J Environ Public Health. 2022;2022:15. 10.1155/2022/4403976 PMC9532122 36203500

[28] Faria AL, Almeida Y, Branco D, Câmara J, Cameirão M, Ferreira L, et al. NeuroAIreh@b: an artificial intelligence-based methodology for personalized and adaptive neurorehabilitation. Front Neurol. 2024;14:14. 10.3389/fneur.2023.1258323 PMC10846403 38322797

[29] Park J-H. Effects of personalized cognitive training using mental workload monitoring on executive function in older adults with mild cognitive impairment. Brain Neurorehabil. 2023;16(3): e21. 38047099 10.12786/bn.2023.16.e21 PMC10689865

[30] Book S, Jank M, Pendergrass A, Graessel E. Individualised computerised cognitive training for community-dwelling people with mild cognitive impairment: study protocol of a completely virtual, randomised, controlled trial. Trials. 2022;23(1):1–13. 35513855 10.1186/s13063-022-06152-9 PMC9069424

[31] Altman DG, Bland JM. Treatment allocation by minimisation. BMJ. 2005;330(7495): 843. 15817555 10.1136/bmj.330.7495.843 PMC556084

[32] R Core Team. R: a language and environment for statistical computing. : R Foundation for Statistical Computing; 2019. Available from: https://www.R-project.org/.

[33] Luttenberger K, Donath C, Uter W, Graessel E. Effects of multimodal nondrug therapy on dementia symptoms and need for care in nursing home residents with degenerative dementia: a randomized-controlled study with 6-month follow-up. J Am Geriatr Soc. 2012;60(5):830–40. 22468985 10.1111/j.1532-5415.2012.03938.x

[34] Graessel E, Stemmer R, Eichenseer B, Pickel S, Donath C, Kornhuber J, et al. Non-pharmacological, multicomponent group therapy in patients with degenerative dementia: a 12-month randomised, controlled trial. BMC Med. 2011;9(1): 129. 22133165 10.1186/1741-7015-9-129 PMC3254071

[35] Nasreddine ZS, Phillips NA, Bedirian V, Charbonneau S, Whitehead V, Collin I, et al. The Montreal Cognitive Assessment, MoCA: a brief screening tool for mild cognitive impairment. J Am Geriatr Soc. 2005;53(4):695–9. 15817019 10.1111/j.1532-5415.2005.53221.x

[36] Freitas S, Simoes MR, Alves L, Santana I. Montreal cognitive assessment: validation study for mild cognitive impairment and Alzheimer disease. Alzheimer Dis Assoc Disord. 2013;27(1):37–43. 22193353 10.1097/WAD.0b013e3182420bfe

[37] Sala G, Inagaki H, Ishioka Y, Masui Y, Nakagawa T, Ishizaki T, et al. The psychometric properties of the Montreal Cognitive Assessment (MoCA). Swiss J Psychol. 2020;79(3–4):155–61.

[38] Freitas S, Prieto G, Simões MR, Santana I. Scaling cognitive domains of the montreal cognitive assessment: an analysis using the partial credit model. Arch Clin Neuropsychol. 2015;30(5):435–47. 25944337 10.1093/arclin/acv027

[39] O’Caoimh R, Timmons S, Molloy DW. Screening for mild cognitive impairment: comparison of “MCI specific” screening instruments. J Alzheimer’s Dis. 2016;51(2):619–29. 26890758 10.3233/JAD-150881 PMC4927818

[40] Ciesielska N, Sokolowski R, Mazur E, Podhorecka M, Polak-Szabela A, Kedziora-Kornatowska K. Is the Montreal Cognitive Assessment (MoCA) test better suited than the Mini-Mental State Examination (MMSE) in mild cognitive impairment (MCI) detection among people aged over 60? Meta-analysis. Psychiatria Polska. 2016;50(5):1039–52. 27992895 10.12740/PP/45368

[41] Thomann AE, Berres M, Goettel N, Steiner LA, Monsch AU. Enhanced diagnostic accuracy for neurocognitive disorders: a revised cut-off approach for the montreal cognitive assessment. Alzheimer’s Res Ther. 2020;12(1):39. 32264975 10.1186/s13195-020-00603-8 PMC7140337

[42] Bruijnen CJWH, Dijkstra BAG, Walvoort SJW, Budy MJJ, Beurmanjer H, De Jong CAJ, et al. Psychometric properties of the Montreal Cognitive Assessment (MoCA) in healthy participants aged 18–70. Int J Psychiatry Clin Pract. 2020;24(3):293–300. 32271127 10.1080/13651501.2020.1746348

[43] Folstein M, Folstein S, Mc HP. “Mini-Mental State”: a practical method for grading the cognitive state of patients for the clinician. J Psychiatr Res. 1975;12(3):189–98. 1202204 10.1016/0022-3956(75)90026-6

[44] Arevalo-Rodriguez I, Smailagic N, Roque IFM, Ciapponi A, Sanchez-Perez E, Giannakou A, et al. Mini-Mental State Examination (MMSE) for the detection of Alzheimer’s disease and other dementias in people with mild cognitive impairment (MCI). Cochrane Database Syst Rev. 2015;3:CD010783. 10.1002/14651858.CD010783.pub2 PMC6464748 25740785

[45] Tombaugh TN, McIntyre NJ. The mini-mental state examination: a comprehensive review. J Am Geriatr Soc. 1992;40(9):922–35. 1512391 10.1111/j.1532-5415.1992.tb01992.x

[46] Creavin ST, Wisniewski S, Noel-Storr AH, Trevelyan CM, Hampton T, Rayment D, et al. Mini-Mental State Examination (MMSE) for the detection of dementia in clinically unevaluated people aged 65 and over in community and primary care populations. Cochrane Database Syst Rev. 2016;2016(1):CD011145. 26760674 10.1002/14651858.CD011145.pub2 PMC8812342

[47] Zhang S, Qiu Q, Qian S, Lin X, Yan F, Sun L, et al. Determining appropriate screening tools and cutoffs for cognitive impairment in the Chinese elderly. Front Psychiatry. 2021;12: 773281. 34925100 10.3389/fpsyt.2021.773281 PMC8674928

[48] Salis F, Costaggiu D, Mandas A. Mini-mental state examination: optimal cut-off levels for mild and severe cognitive impairment. Geriatrics. 2023;8(1): 12. 36648917 10.3390/geriatrics8010012 PMC9844353

[49] Kroenke K, Spitzer RL, Williams JBW. The PHQ-9: validity of a brief depression severity measure. J Gen Intern Med. 2001;16(9):606–13. 11556941 10.1046/j.1525-1497.2001.016009606.x PMC1495268

[50] Kroenke K, Spitzer RL, Williams JBW, Löwe B. The patient health questionnaire somatic, anxiety, and depressive symptom scales: a systematic review. Gen Hosp Psychiatry. 2010;32(4):345–59. 20633738 10.1016/j.genhosppsych.2010.03.006

[51] Gilbody S, Richards D, Barkham M. Diagnosing depression in primary care using self-completed instruments: UK validation of PHQ-9 and CORE-OM. Br J Gen Pract. 2007;57(541):650–2. 17688760 PMC2099671

[52] Lippert T, Maas R, Fromm MF, Luttenberger K, Kolominsky-Rabas P, Pendergrass A, et al. Impact of sedating drugs on falls resulting injuries among people with dementia in a nursing home setting. Gesundheitswesen. 2020;82(1):14–22. 31962367 10.1055/a-1071-7911

[53] Quan H, Li B, Couris CM, Fushimi K, Graham P, Hider P, et al. Updating and validating the Charlson comorbidity index and score for risk adjustment in hospital discharge abstracts using data from 6 countries. Am J Epidemiol. 2011;173(6):676–82. 21330339 10.1093/aje/kwq433

[54] Graessel E, Jank M, Greiner S, Stemmler M. First validation of the Computerized Cognitive Test Battery (CCTB) on the Short Cognitive Performance Test (SKT) and the Montreal Cognitive Assessment (MoCA) in terms of convergent and divergent validity in individuals aged 60 years and older. Erlangen: Open FAU; 2024. 77 p.

[55] Wechsler D. Wechsler adult intelligence scale–fourth edition (WAIS-IV)–Deutsche Version. Petermann F, editor. Frankfurt/Main: Pearson Assessment; 2012.

[56] Lehfeld H, Schläfke S, Hoerr R, Stemmler M. SKT short cognitive performance test and activities of daily living in dementia. Geropsych (Bern). 2014;27(2):75–80.

[57] Henik A, Tzelgov J. Is three greater than five: the relation between physical land semantic size in comparison tasks. Mem Cognition. 1982;10(4):389–95. 10.3758/bf03202431 7132716

[58] Raven JC, Court JH. Manual for Raven’s progressive matrices and vocabulary scales. London: H.K. Lewis; 1986.

[59] Laugwitz B, Held T, Schrepp M, editors. Construction and evaluation of a User Experience Questionnaire. Berlin, Heidelberg: Springer Berlin Heidelberg; 2008.

[60] Schrepp M. User Experience Questionnaire Handbook. 2023. 16 p.

[61] Cohen J. Statistical power analysis for the behavioral sciences. 2nd ed. Hillsdale: Erlbaum Associates; 1988.

[62] Yu J-T, Xu W, Tan C-C, Andrieu S, Suckling J, Evangelou E, et al. Evidence-based prevention of Alzheimer’s disease: systematic review and meta-analysis of 243 observational prospective studies and 153 randomised controlled trials. J Neurol Neurosurg Psychiatry. 2020;91(11):1201–9. 32690803 10.1136/jnnp-2019-321913 PMC7569385

[63] Field A. Discovering statistics using IBM SPSS Statistics. 6 th ed. London: SAGE Publications Limited; 2024.

[64] Pituch KA, Stevens JP. Applied multivariate statistics for the social sciences: analyses with SAS and IBM’s SPSS. 6th ed. New York: Routledge; 2015. p. 814.

[65] International Council for Harmonisation of Technical Requirements for Pharmaceuticals for Human Use (ICH). ICH: E 9: Statistical principles for clinical trials - Step 5 - Note for guidance on statistical principles for clinical trials (CPMP/ICH/363/96). European Medicines Ageny; 1998. Available from: https://www.ema.europa.eu/en/ich-e9-statistical-principles-clinical-trials-scientific-guideline#current-version-8451.

[66] World Health Organization. ICD-10: international statistical classification of diseases and related health problems: tenth revision. Geneva: World Health Organization; 2004.

[67] Canevelli M, Adali N, Voisin T, Soto M, Bruno G, Cesari M, et al. Behavioral and psychological subsyndromes in Alzheimer’s disease using the Neuropsychiatric Inventory. Int J Geriatr Psychiatry. 2013;28(8):795–803. 23147419 10.1002/gps.3904

[68] German Federal Statistical Office. Verteilung der Bevölkerung in Deutschland nach höchstem Schulabschluss im Jahr 2023: Statista; 2024. Available from: https://de.statista.com/statistik/daten/studie/1988/umfrage/bildungsabschluesse-in-deutschland/.

[69] Thomann AE, Goettel N, Monsch RJ, Berres M, Jahn T, Steiner LA, et al. The montreal cognitive assessment: normative data from a German-speaking cohort and comparison with international normative samples. J Alzheimer’s Dis. 2018;64(2):643–55. 29945351 10.3233/JAD-180080 PMC6027948

[70] Pinto TC, Machado L, Bulgacov TM, Rodrigues-Júnior AL, Costa ML, Ximenes RC, et al. Is the Montreal Cognitive Assessment (MoCA) screening superior to the Mini-Mental State Examination (MMSE) in the detection of mild cognitive impairment (MCI) and Alzheimer’s Disease (AD) in the elderly? Int Psychogeriatr. 2019;31(4):491–504. 30426911 10.1017/S1041610218001370

[71] Tan JP, Li N, Gao J, Wang LN, Zhao YM, Yu BC, et al. Optimal cutoff scores for dementia and mild cognitive impairment of the montreal cognitive assessment among elderly and oldest-old Chinese population. J Alzheimer’s Dis. 2015;43(4):1403–12. 25147113 10.3233/JAD-141278

[72] Berg J-L, Durant J, Leger GC, Cummings JL, Nasreddine Z, Miller JB. Comparing the electronic and standard versions of the montreal cognitive assessment in an outpatient memory disorders clinic: a validation study. J Alzheimers Dis. 2018;62(1):93–7. 29439349 10.3233/JAD-170896 PMC5817908

[73] Zeng B, Tang C, Wang J, Yang Q, Ren Q, Liu X. Pharmacologic and nutritional interventions for early Alzheimer’s disease: a systematic review and network meta-analysis of randomized controlled trials. J Alzheimer’s Dis. 2024(Preprint):1–14. 10.3233/JAD-240161 PMC11191524 38759015

